# Nonsteroidal anti-inflammatory drugs using and risk of head and neck cancer: a dose–response meta analysis of prospective cohort studies

**DOI:** 10.18632/oncotarget.21524

**Published:** 2017-10-05

**Authors:** Jun Shi, Weidong Leng, Lunhua Zhao, Chenli Xu, Jue Wang, Xiaoli Chen, Yu Wang, Xingchun Peng

**Affiliations:** ^1^ Department of Stomatology, Taihe Hospital, Hubei University of Medicine, Shiyan, Hubei, 442000, China; ^2^ School of Basic Medical Sciences, Hubei University of Medicine, Shiyan, Hubei, 442000, China; ^3^ Department of Ultrasonography, Xiangyang No.1 People's Hospital, Hubei University of Medicine, Xiangyang, Hubei, 441000, China; ^4^ Department of Oncology, Suizhou Hospital, Hubei University of Medicine, Suizhou, Hubei, 441300, China

**Keywords:** head and neck cancer, nonsteroidal anti-inflammatory drugs, chemoprevention, meta analysis

## Abstract

Conflicting results identifying the relationship between nonsteroidal anti-inflammatory drugs using and head and neck cancer risk. Therefore, we performed this meta-analysis to clarify and quantitative assessed the relationship between nonsteroidal anti-inflammatory drugs using and head and neck cancer risk. Up to March 2017, 11 original publications were included in this meta-analysis. Our results showed statistically significant association between nonsteroidal anti-inflammatory drugs using and head and neck cancer risk reduction. Subgroups analysis indicated that Aspirin, COX 2 inhibitors, Ibuprofen and Other NSAIDs were associated with a significantly risk reduction of head and neck cancer. Furthermore, nonsteroidal anti-inflammatory drugs using was associated with a significantly lower risk of oral and oropharynx cancer, larynx cancer and hypopharynx cancer. In addition, increasing nonsteroidal anti-inflammatory drugs using (per 2 prescriptions/week increment) was associated with a 4% reduction in head and neck cancer risk, 5% reduction of aspirin using and 6% reduction of other nonsteroidal anti-inflammatory drugs using. Considering these promising results, increasing nonsteroidal anti-inflammatory drugs using might provide health benefits. More studies and large sample size are warranted to validate this association.

## INTRODUCTION

Head and neck cancer (HNC) include tumors derived from any tissue or organ from the eyes, brain, ears, thyroid and esophageal head and neck, and over 90% of HNC are squamous cell carcinoma. The incidence of global head and neck squamous cell carcinoma (SCCHN) has increased significantly over the last 10 years, especially in women. There are about 645,000 new cases of HNC occurring each year. In 2002, the new cases of HNC was about 100800 cases in Europe, and cause more than 40,000 deaths [1]. The etiology of Head and neck cancer involves both genetic and environmental factors. Tobacco consumption and excessive alcohol drinking are independent major risk factors for HNC development [2–4]. Despite significant advances in multidisciplinary treatment, about 30–50% of HNC patients survived for 5 years [5, 6]. Therefore, further studies should be conducted to identify potential chemical precautions other than tobacco alcohol abstinence.

Non-steroidal antiinflammatory drugs NSAIDs (NSAIDs) have been widely used in clinical. Since the 1980s, NSAIDs have received widespread attention as anti-tumor properties. Such drugs include aspirin, acetaminophen, indomethacin, naproxen, naproxen, diclofenac, ibuprofen, nimesulide, rofecoxib and celecoxib. With anti-inflammatory, anti-rheumatic, pain, fever and anticoagulation and other effects, widely used in clinical osteoarthritis, rheumatoid arthritis, a variety of fever and various pain symptoms [7].

Previous studies have examined the relationship between NSAIDs use and risk of colon cancer, stomach cancer, prostate cancer and breast cancer, have found that NSAIDs use is significantly reduce cancer risk [8–11]. However, no study to clarify and quantitative assessed NSAIDs use in relation to HNC risk. Thus, we conducted this dose-response meta-analysis to clarify and quantitative assessed the relationship between NSAIDs use and HNC risk.

## MATERIALS AND METHODS

Our meta-analysis was conducted according to the Meta-analysis Of Observational Studies in Epidemiology (MOOSE) checklist [12].

### Search strategy

We included eligible studies investigating the relationship of NSAIDs using and HNC risk in general adult populations. To develop a flexible, non-linear, r meta-regression model, we required that an eligible study should have categorized NSAIDs duration into 3 or more levels. If multiple publications were available for a study, we included the longest follow-up study.

Eligible studies were systematically searched of PubMed and Embase update to March 2017 for case control or cohort studies examining the relationship between NSAIDs using and HNC risk, with keywords including “head and neck cancer” OR”HNC”OR “Oral” OR “Oropharynx” OR “hypopharynx” OR “larynx” AND “aspirin” OR “NSAIDs” OR “ibuprofen” OR “naproxen” OR “indomethacin” OR “meloxicam” OR “valdecoxib” OR “celecoxib” OR “rofecoxib”. We refer to the relevant original essays and commentary articles to determine further relevant research. Eligible study was included through the reference lists of relevant review articles.

### Study selection

Two independent researchers investigate information the relationship between NSAIDs use and HNC risk: study design in case-control or cohort study; outcome was head and neck cancer; the relative risks at least three quantitative categories of NSAIDs use and head and neck cancer risk with 95% confidence intervals. Moreover, we precluded non-human studies, reviews, editorials and published letters. The disagreements were resolved through consensus by all of the authors.

### Data extraction

Use standardized data collection tables to extract data. The two researchers extracted detailed information from each included article. We extracted the following information: first author; publication year; age; country; sex; cases and participants; the categories of NSAIDs use; relative risk or odds ratio (OR) with 95% confidence intervals. We collect the risk estimates with multivariable-adjusted [13]. Quality assessment was performed according to the Newcastle-Ottawa scale for non-randomized studies [14].

### Statistical analysis

We pooled relative risk estimates as the common measure of association NSAIDs use and head and neck cancer risk; the hazard ratio were considered equivalent to the relative risk [15]. Results in different subgroups of NSAIDs using and HNC risk were treated as two separate reports.

Due to different cut-off points in the included studies for categories, we performed a relative risk with 95% confidence interval by increase per 2 prescriptions/week using the method recommended by Greenland, Longnecker and Orsini and their colleagues. Dose of NSAIDs using used the median NSAIDs using. If the median NSAIDs using category was not available, the midpoint of the upper and lower boundaries was considered the dose of each category. The midpoint of the category was set at 1.5 times the lower boundary if the highest category was open ended. We use the midpoint of the category if the highest and lowest values are not reported. The midpoint of the category is estimated by assuming that the width of the category is the same as the next adjacent category if the highest or lowest category is open. In addition, we evaluated the non-linear association between NSAIDs using and head and neck cancer risk using restricted cubic splines, with three knots at the 10th, 50th, and 90th percentiles of the distribution. A flexible meta-regression based on restricted cubic spline (RCS) function was used to fit the potential non-linear trend, and generalized least-square method was used to estimate the parameters. This procedure treats nonsteroidal anti-inflammatory drugs using (continuous data) as an independent variable and logRR of diseases as a dependent variable, with both tails of the curve restricted to nonlinear. A *P* value is calculated for linear or non-linear by testing the null hypothesis that the coefficient of the second spline is equal to zero [16].

The between-study heterogeneity was assessed by Q-statistic and the I^2^-statistic. All analyses were conducted using STATA software 12.0 (STATA Corp, College Station, TX, USA). *P* < 0.05 was considered significant for all tests.

## RESULTS

### Literature search results

**Figure 1:**
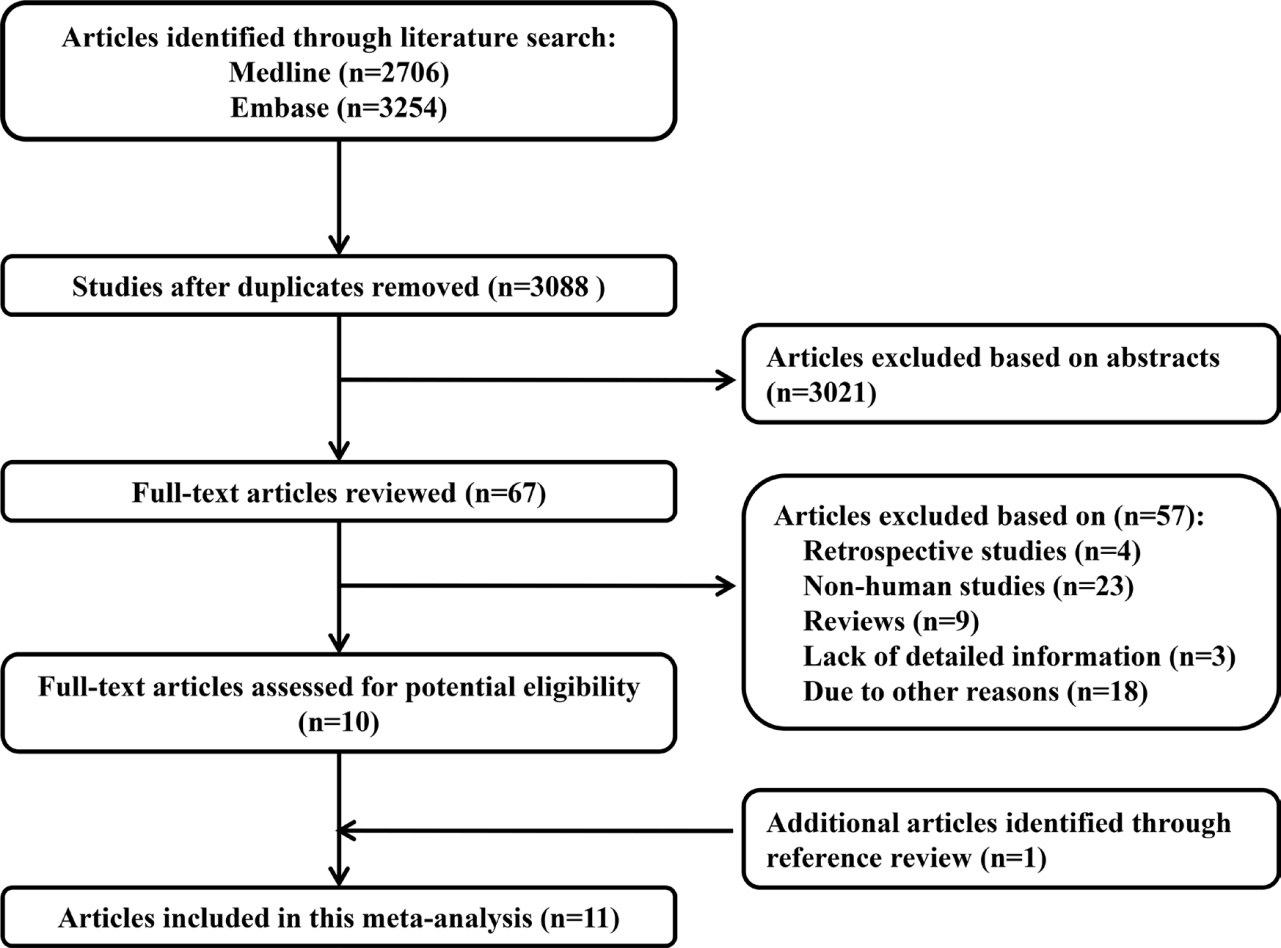
Flow diagram of the study selection process

We identifed 3088 relevant citations after exclusion of duplicates. After exclusion studies that did not fulfill the inclusion criteria, eleven studies were chosen, and the data were extracted. Results in different subgroups of NSAIDs using and head and neck cancer risk were treated as two separate reports, a total of 33 reports data were included in this meta-analysis. These studies were published update to March 2017. Figure 1 shows the results of literature research and selection.

### Study characteristics

The characteristics of the included studies of nonsteroidal anti-inflammatory drugs using and risk of head and neck cancer are shown in the Tables 1 and 2. Among the selected studies, four cohort studies [17–20] and seven case–control studies [6, 21–26], a total of 653828 participants with 12637 incident cases were included in this meta-analysis.

**Table 1:** Characteristics of participants in included studies of nonsteroidal anti-inflammatory drugs using and risk of head and neck cancer

Author (year)	Study name	Study design	Country	Sex of population	Age at baseline (years)	No of participants	No of cases	Quality score
Ahmadi et al. (2010)	The Lombardi Comprehensive Cancer Center	Case-control	USA	Mix	56	142	71	5
Becker et al. (2015)	CPRD	Case–control	UK	Mix	< 90	19215	2745	7
Bosetti et al. (2003)	NA	Case–control	Italy	Mix	> 25	2744	965	6
Di et al. (2015)	NA	Case–control	Caucasian	Mix	52	790	198	6
Friis et al. (2006)	The Danish Civil Registration System	Cohort	Denmark	Mix	> 16	442654	260	8
Friis et al. (2003)	The Danish Civil Registration System	Cohort	Denmark	Mix	> 16	29470	2187	8
Jayaprakash et al. (2006)	The Roswell Park Cancer Institute	Case–control	USA	Mix	40	1058	529	6
Macfarlane et al. (2012)	The ARCAGE study	Case–control	Europe	Mix	> 25	3772	1779	7
Macfarlane et al. (2014)	PCCIU	Case–control	Scotland	Mix	66	9557	2392	7
Macfarlane et al. (2015)	PCCIU	Cohort	Scotland	Mix	66	2392	1195	7
Wilson et al. (2013)	The PLCO trial	Cohort	USA	Mix	55–74	142034	316	7

CPRD: The UK-based Clinical Practice Research Datalink; ARCAGE: The Alcohol-Related CAncers and GEnetic Susceptibility; PCCIU: The Primary CareClinical Informatics Unit; The PLCO trial: The United States National Cancer Institute Prostate, Lung, Colorectal and Ovarian Cancer Screening Trial

**Table 2:** Outcomes and covariates of included studies of nonsteroidal anti-inflammatory drugs using and risk of head and neck cancer

Author (year)	Endpoints	Data source	Category and relative risk (95% CI)	Covariates in fully adjusted model
Becker et al. (2015)		Population-based	COX 2 inhibitors No prior use, 1.0 (reference);1 RX, 1.14 (0.89, 1.47); > 2– < 5, 1.0 (0.73, 1.35); ; ≥ 6, 1.12 (0.81, 1.55) Diclofenac No prior use, 1.0 (reference);1 RX, 0.97 (0.86, 1.10); > 2– < 5, 1.02 (0.89, 1.17); ; ≥ 6, 1.05 (0.89, 1.25) Ibuprofen No prior use, 1.0 (reference);1 RX, 1.00 (0.89, 1.12); > 2– < 5, 0.94 (0.83, 1.08); ; ≥ 6, 0.78 (0.64, 0.96) Naproxen No prior use, 1.0 (reference);1 RX, 0.97 (0.81, 1.15); > 2– < 5, 0.97 (0.72, 1.14); ; ≥ 6, 1.18 (0.88, 1.58) Other NSAIDs No prior use, 1.0 (reference);1 RX, 0.97 (0.81, 1.15); > 2– < 5, 1.15 (0.95, 1.38); ; ≥ 6, 0.99 (0.80, 1.22) Aspirin No prior use, 1.0 (reference);1 RX, 1.10 (0.94, 1.28); > 2– < 5, 1.02 (0.88, 1.18); ; ≥ 6, 1.21 (0.97, 1.51)	Adjusted for all other medications in this table, Body mass index, smoking, and alcohol consumption
Friis et al. (2006)		Self-administered	Aspirin No prior use, 1.0 (reference); > 2– < 9, 1.0 (0.6, 1.6); ; ≥ 10, 1.31 (1.0,1.6)	Adjusted for age, sex
Jayaprakash et al. (2006)		Population-based	Aspirin No prior use, 1.0 (reference);1 RX, 0.68 (0.46, 1.02); > 2– < 6, 1.08 (0.75, 1.54); ; ≥ 7, 0.68(0.47, 0.99)	Adjusted for age, sex, packs of cigarettes per day, and alcoholic drinks per week
Macfarlane et al. (2012)		Population-based	Aspirin No prior use, 1.0 (reference);1 RX, 0.98 (0.72, 1.34); > 2– < 6, 0.80 (0.62, 1.04); ; ≥ 7, 0.96(0.75, 1.25)	Adjusted for centre, age, gender, education, smoking (pack-years), alcohol drinking (drink-years), fruit consumption and body mass index 2 years ago
Macfarlane et al. (2014)		Self-administered	Aspirin No prior use, 1.0 (reference); > 0.4– < 4, 0.88 (0.70, 1.09); > 5– < 7, 0.82 (0.67, 1.00); > 8– < 40, 0.90 (0.75, 1.25)	adjusted for deprivation, BMI (o25), smoking (ever), alcohol consumption (high), CHD, stroke
Macfarlane et al. (2015)		Self-administered	Aspirin No prior use, 1.0 (reference); < 3, 0.81 (0.60, 1.09); > 3– < 6, 0.48 (0.33, 0.71); > 6, 0.55(0.36, 0.84)	Adjusted for age, gender, deprivation, year of diagnosis, smoking (ever), alcohol consumption (high), CHD, AF, Stroke, Aspirin ((before and after diagnosis), COX-2 (before and after diagnosis), other NSAID (before and after diagnosis) and taking into account clustering within medical practices.
Wilson et al. (2013)		Self-administered	Aspirin No prior use, 1.0 (reference); 1, 0.69 (0.51, 0.93); 7, 0.85 (0.65, 1.11) Ibuprofen No prior use, 1.0 (reference); 1, 1.03 (0.76, 1.39); 7, 0.86 (0.56, 1.32)	Multivariate adjustments: age at baseline (years), gender, BMI (o18.5, 18.5–o25, 25–o30, X30 kg m 2), tobacco use (None, 40–29, 429–49, 449 maximum cigarette pack years); Ibuprofen model further adjusted for aspirin use

Rx = prescription.

### Overall meta-analysis

The results of NSAIDs using and HNC risk are shown in Table 3. The pooled results suggest that NSAIDs using is significantly associated with head and neck cancer risk, which was suggested both by the highest NSAIDs using versus lowest NSAIDs using (RR:0.84; 95% CI, 0.76–0.93; *P* < 0.001) (Table 3). We found evidence of between-study heterogeneity (I^2^ = 70.5%, *P* = 0.000) but we observed no evidence of publication bias (Egger asymmetry test, *P* = 0.245) (Supplementary Table 2).

**Table 3:** Stratified analyses of relative risk of head and neck cancer

	No of reports	Relative risk (95% CI)	P for heterogeneity	I2	*P* for test
**Type of drugs use**					
Total	33	0.84 (0.76–0.93)	0.000	70.5%	*P* < 0.01
Aspirin Use	22	0.85 (0.74–0.96)	0.000	66.0%	*P* < 0.01
COX 2 inhibitors	3	0.79 (0.70–0.98)	0.357	3.0%	*P* < 0.01
Ibuprofen	2	0.85 (0.69–0.97)	0.223	32.8%	*P* < 0.01
Other NSAIDs	6	0.76 (0.59–0.94)	0.000	88.2%	P < 0.01
**HNC sites**					
Oral and oropharynx	6	0.85 (0.77–0.94)	0.118	43.0%	*P* < 0.01
Larynx	3	0.76 (0.66–0.92)	0.155	46.3%	*P* < 0.01
Hypopharynx	2	0.59 (0.27–0.91)	0.532	0.0%	*P* < 0.01
**Study design**					
Cohort	8	0.85 (0.72–0.98)	0.000	76.7%	*P* < 0.01
Case-control	25	0.83 (0.73–0.93)	0.000	68.5%	*P* < 0.01
**No of participants**					
≥ 10 000	11	0.82 (0.71–0.93)	0.014	55.1%	*P* < 0.01
< 10 000	22	0.74 (0.64–0.83)	0.000	64.6%	*P* < 0.01
**No of cases**					
≥ 500	28	0.84 (0.75–0.93)	0.000	70.0%	*P* < 0.01
< 500	5	0.76 (0.58–0.98)	0.001	77.9%	*P* < 0.01
**Study quality**					
Score ≥ 7	23	0.91 (0.83–0.99)	0.000	64.9%	*P* < 0.01
Score < 7	10	0.60 (0.40–0.80)	0.002	65.5%	*P* < 0.01

*P* for test: The test for highest versus lowest meta-analysis on drugs use and head and neck cancer risk.

### Dose–response meta-analyses between NSAIDs using and head and neck cancer

**Figure 2:**
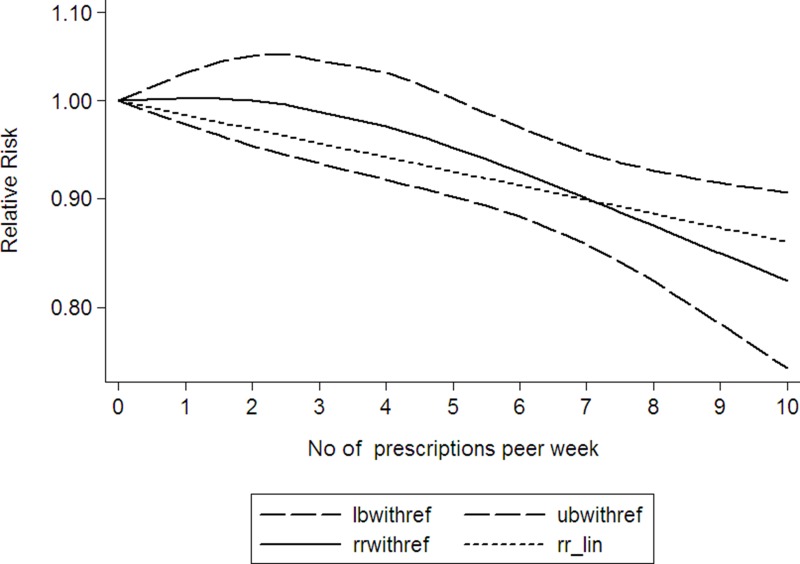
Dose-response relationship between NSAIDs using and head and neck cancer (The solid line represents fitted non-linear trend, the dotted line represents the 95% confdence interval).

**Figure 3:**
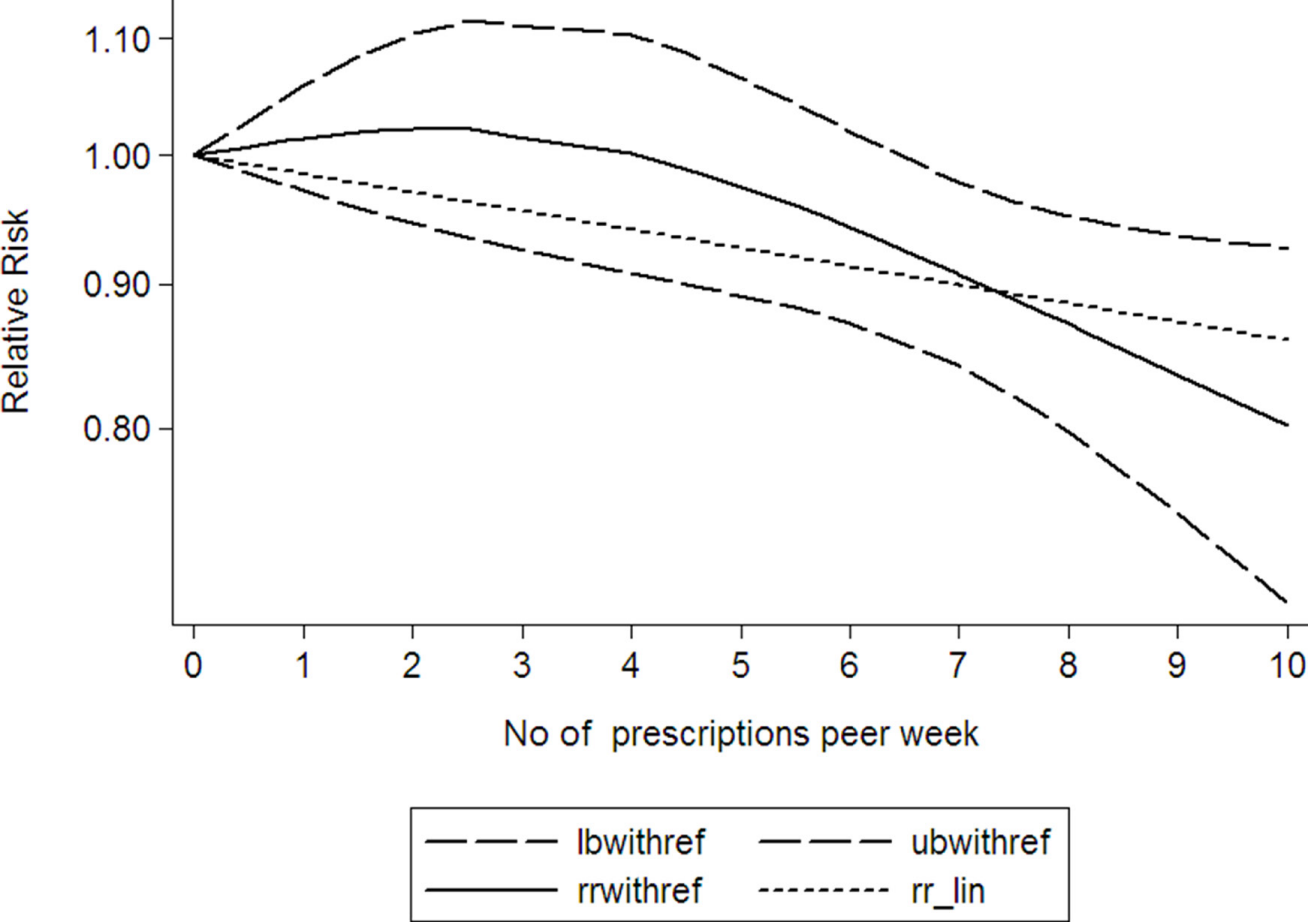
Dose-response relationship between aspirin using and head and neck cancer (The solid line represents fitted non-linear trend, the dotted line represents the 95% confdence interval).

**Figure 4:**
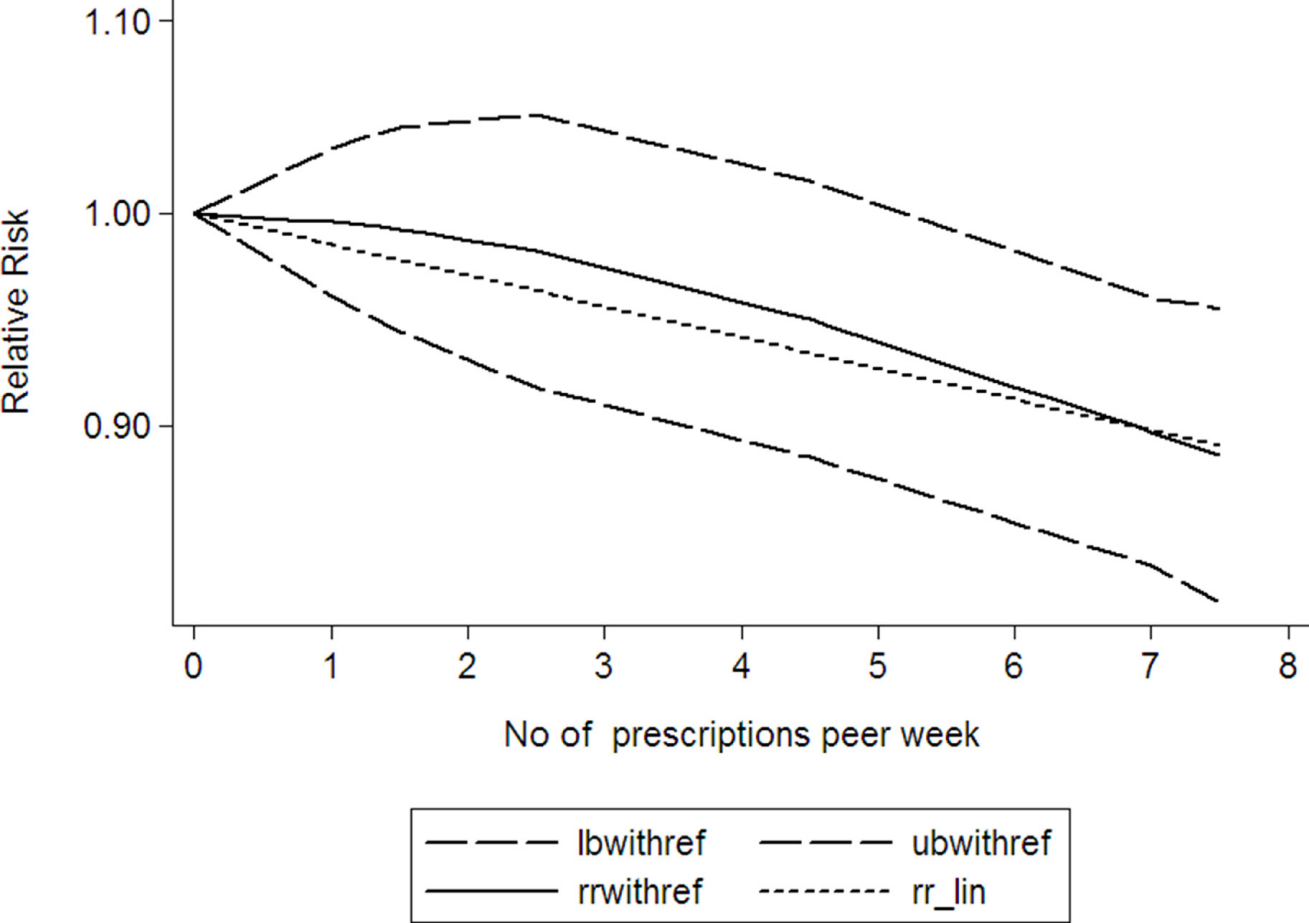
Dose-response relationship between other NSAIDs using and head and neck cancer (The solid line represents fitted non-linear trend, the dotted line represents the 95% confdence interval).

Using restricted cubic spline function, the test for a nonlinear dose-response relationship was significant (likelihood ratio test, *P* = 0.000), suggesting curvature in the relationship, increase per 2 prescriptions/week of NSAIDs using was associated with a 4% decremental in head and neck cancer risk, the summary relative risk of head and neck cancer risk for an increase per 2 prescriptions/week of NSAIDs using was 0.96 (95% CI: 0.94–0.99, *P* < 0.001) (Figure 2). Increasing aspirin using (per 2 prescriptions/week increment) was related to a 5% reduction in head and neck cancer risk (RR: 0.95; 95% CI, 0.91–0.99) (Figure 3). Increasing other NSAIDs using (per 2 prescriptions/week increment) was related to a 6% reduction in head and neck cancer risk (RR: 0.94; 95% CI, 0.89–0.96) (Figure 4).

### Subgroup analyses

Subgroup analysis was performed to check the stability of the primary outcome (Table 3). Subgroups analysis indicated that Aspirin (RR:0.85; 95% CI, 0.74–0.96; *P* < .001) (Table 3), COX 2 inhibitors (RR:0.79; 95% CI, 0.70–0.98; *P* < .001) (Table 3), Ibuprofen(RR:0.85; 95% CI, 0.69–0.97; *P* < .001) (Table 3) and Other NSAIDs (RR:0.76; 95% CI, 0.59–0.94; *P* < .001) (Table 3) were associated with a significantly risk of head and neck cancer. Furthermore, nonsteroidal anti-inflammatory drugs using was associated with a significantly lower risk of Oral and oropharynx cancer (RR:0.85; 95% CI, 0.77–0.94; *P* < .001) (Table 3), Larynx cancer (RR:0.76; 95% CI, 0.66–0.92; *P* < .001) (Table 3) and Hypopharynx cancer (RR:0.59; 95% CI, 0.27–0.91; *P* < .001) (Table 3).

### Sensitivity analysis

A sensitivity analysis omitting 1 study at a time and calculating the pooled ORs for the remainder of the studies showed that 2 studies by Becker et al. (2015) [22] and Macfarlane et al. (2012) [25] substantially influenced the pooled OR. After excluding the 2 studies, there was no study heterogeneity (PQ = 0.965, I^2^ = 0.0%), and the OR for the highest vs lowest category of NSAIDs using was 0.83 (95% CI, 0.75–0.96) (Supplementary Table 1).

### Publication bias

The results show no obvious evidence of publication bias was found in the associations between NSAIDs using and head and neck cancer risk (Supplementary Table 2).

## DISCUSSION

NSAIDs as antipyretic analgesic anti-inflammatory drugs have been widely used in clinical, especially in the prevention and treatment of cardiovascular disease. Since the 80s of last century, NSAIDs anti-tumor properties have been widespread concern. Vinogradova found that long-term using of NSAIDs and cyclooxygenase-2 (COX-2) inhibitors can reduce the risk of developing colorectal cancer through a large sample of clinical case-control analysis [27]. Another study shows that NSAIDs can reduce the incidence of colonic adenoma and postoperative recurrence rate [28, 29]. Moreover, NSAIDs can reduce esophageal cancer, endometrial cancer, ovarian cancer, prostate cancer, bladder cancer, malignant melanoma, skin cancer and other malignant tumors risk [30–36], but since then, studies of NSAIDs in head and neck cancer increased slowly.

Among the eleven included studies, eight showed a signifcantly positive association between NSAIDs using and head and neck cancer risk, and have found that NSAIDs use is significantly reduce head and neck cancer risk, while the other were negative. Studies on different NSAIDs using and head and neck cancer risk also remain inconclusive in light of newer evidence.

To our knowledge, this is the first study to identify and quantify the potential dose-response association between NSAIDs using and head and neck cancer in a large cohort of both men and women. The primary finding in our meta-analysis is that statistically significant association between nonsteroidal anti-inflammatory drugs using and head and neck cancer. Subgroups analysis indicated that Aspirin, COX 2 inhibitors, Ibuprofen and Other NSAIDs using were associated with a significantly risk of head and neck cancer. Furthermore, nonsteroidal anti-inflammatory drugs using was associated with a significantly lower risk in oral and oropharynx cancer, larynx cancer and hypopharynx cancer. In addition, increasing nonsteroidal anti-inflammatory drugs using (per 2 prescriptions/week increment) was associated with a 4% reduction in head and neck cancer risk, 5% reduction of aspirin using and 6% reduction of other nonsteroidal anti-inflammatory drugs using.

Although, we performed this meta-analysis very carefully, some limitations must be considered in the current meta-analysis. Firstly, different sex of population should be included in this meta-analysis to explore the impact of different sex of population on nonsteroidal anti-inflammatory drugs using and head and neck cancer. Secondly, despite we searched all studies describing the association between NSAIDs using and HNC risk, there are only 11 studies about NSAIDs using and HNC risk, the number of eligible studies was still limited.

In conclusion, our meta-analysis suggests NSAIDs using was independently associated with deleterious head and neck cancer risk reduction. However, more studies and large sample size are warranted to validate this association.

## SUPPLEMENTARY MATERIALS FIGURES AND TABLES



## References

[1] Ferlay J, Soerjomataram I, Dikshit R, Eser S, Mathers C, Rebelo M, Parkin DM, Forman D, Bray F (2015). Cancer incidence and mortality worldwide: sources, methods and major patterns in GLOBOCAN 2012. Int J Cancer.

[2] Hashibe M, Brennan P, Benhamou S, Castellsague X, Chen C, Curado MP, Dal Maso L, Daudt AW, Fabianova E, Fernandez L, Wunsch-Filho V, Franceschi S, Hayes RB (2007). Alcohol drinking in never users of tobacco, cigarette smoking in never drinkers, and the risk of head and neck cancer: pooled analysis in the International Head and Neck Cancer Epidemiology Consortium. J Natl Cancer Inst.

[3] Petti S (2009). Lifestyle risk factors for oral cancer. Oral Oncol.

[4] Lagiou P, Talamini R, Samoli E, Lagiou A, Ahrens W, Pohlabeln H, Benhamou S, Bouchardy C, Slamova A, Schejbalova M, Merletti F, Richiardi L, Kjaerheim K (2009). Diet and upper-aerodigestive tract cancer in Europe: the ARCAGE study. Int J Cancer.

[5] Khuri FR, Kim ES, Lee JJ, Winn RJ, Benner SE, Lippman SM, Fu KK, Cooper JS, Vokes EE, Chamberlain RM, Williams B, Pajak TF, Goepfert H (2001). The impact of smoking status, disease stage, and index tumor site on second primary tumor incidence and tumor recurrence in the head and neck retinoid chemoprevention trial. Cancer Epidemiol Biomarkers Prev.

[6] Jayaprakash V, Rigual NR, Moysich KB, Loree TR, Nasca MA, Menezes RJ, Reid ME (2006). Chemoprevention of head and neck cancer with aspirin: a case-control study. Arch Otolaryngol Head Neck Surg.

[7] Tanaka T, Nishikawa A, Mori Y, Morishita Y, Mori H (1989). Inhibitory effects of non-steroidal anti-inflammatory drugs, piroxicam and indomethacin on 4-nitroquinoline 1-oxide-induced tongue carcinogenesis in male ACI/N rats. Cancer Lett.

[8] Thun MJ, Henley SJ, Patrono C (2002). Nonsteroidal anti-inflammatory drugs as anticancer agents: mechanistic, pharmacologic, and clinical issues. J Natl Cancer Inst.

[9] Mahmud SM, Franco EL, Aprikian AG (2010). Use of nonsteroidal anti-inflammatory drugs and prostate cancer risk: a meta-analysis. Int J Cancer.

[10] Luo T, Yan HM, He P, Luo Y, Yang YF, Zheng H (2012). Aspirin use and breast cancer risk: a meta-analysis. Breast Cancer Res Treat.

[11] Wang WH, Huang JQ, Zheng GF, Lam SK, Karlberg J, Wong BC (2003). Non-steroidal anti-inflammatory drug use and the risk of gastric cancer: a systematic review and meta-analysis. J Natl Cancer Inst.

[12] Stroup DF, Berlin JA, Morton SC, Olkin I, Williamson GD, Rennie D, Moher D, Becker BJ, Sipe TA, Thacker SB (2000). Meta-analysis of observational studies in epidemiology: a proposal for reporting. Meta-analysis Of Observational Studies in Epidemiology (MOOSE) group. JAMA.

[13] Durrleman S, Simon R (1989). Flexible regression models with cubic splines. Stat Med.

[14] Stang A (2010). Critical evaluation of the Newcastle-Ottawa scale for the assessment of the quality of nonrandomized studies in meta-analyses. Eur J Epidemiol.

[15] Xu C, Zeng XT, Liu TZ, Zhang C, Yang ZH, Li S, Chen XY (2015). Fruits and vegetables intake and risk of bladder cancer: a PRISMA-compliant systematic review and dose-response meta-analysis of prospective cohort studies. Medicine (Baltimore).

[16] Orsini N, Li R, Wolk A, Khudyakov P, Spiegelman D (2012). Meta-analysis for linear and nonlinear dose-response relations: examples, an evaluation of approximations, and software. Am J Epidemiol.

[17] Friis S, Poulsen A, Pedersen L, Baron JA, Sorensen HT (2006). Use of nonsteroidal anti-inflammatory drugs and risk of oral cancer: a cohort study. Br J Cancer.

[18] Friis S, Sorensen HT, McLaughlin JK, Johnsen SP, Blot WJ, Olsen JH (2003). A population-based cohort study of the risk of colorectal and other cancers among users of low-dose aspirin. Br J Cancer.

[19] Macfarlane TV, Murchie P, Watson MC (2015). Aspirin and other non-steroidal anti-inflammatory drug prescriptions and survival after the diagnosis of head and neck and oesophageal cancer. Cancer Epidemiol.

[20] Wilson JC, Murray LJ, Hughes CM, Black A, Anderson LA (2013). Non-steroidal anti-inflammatory drug and aspirin use and the risk of head and neck cancer. Br J Cancer.

[21] Ahmadi N, Goldman R, Seillier-Moiseiwitsch F, Noone AM, Kosti O, Davidson BJ (2010). Decreased risk of squamous cell carcinoma of the head and neck in users of nonsteroidal anti-inflammatory drugs. Int J Otolaryngol.

[22] Becker C, Wilson JC, Jick SS, Meier CR (2015). Non-steroidal anti-inflammatory drugs and the risk of head and neck cancer: A case-control analysis. Int J Cancer.

[23] Bosetti C, Talamini R, Franceschi S, Negri E, Garavello W, La Vecchia C (2003). Aspirin use and cancers of the upper aerodigestive tract. Br J Cancer.

[24] Di Maso M, Bosetti C, La Vecchia C, Garavello W, Montella M, Libra M, Serraino D, Polesel J (2015). Regular aspirin use and nasopharyngeal cancer risk: A case-control study in Italy. Cancer Epidemiol.

[25] Macfarlane TV, Macfarlane GJ, Thakker NS, Benhamou S, Bouchardy C, Ahrens W, Pohlabeln H, Lagiou P, Lagiou A, Castellsague X, Agudo A, Slamova A, Plzak J (2012). Role of medical history and medication use in the aetiology of upper aerodigestive tract cancers in Europe: the ARCAGE study. Ann Oncol.

[26] Macfarlane TV, Lefevre K, Watson MC (2014). Aspirin and non-steroidal anti-inflammatory drug use and the risk of upper aerodigestive tract cancer. Br J Cancer.

[27] Vinogradova Y, Hippisley-Cox J, Coupland C, Logan RF (2007). Risk of colorectal cancer in patients prescribed statins, nonsteroidal anti-inflammatory drugs, and cyclooxygenase-2 inhibitors: nested case-control study. Gastroenterology.

[28] Friis S, Poulsen AH, Sorensen HT, Tjonneland A, Overvad K, Vogel U, McLaughlin JK, Blot WJ, Olsen JH (2009). Aspirin and other non-steroidal anti-inflammatory drugs and risk of colorectal cancer: a Danish cohort study. Cancer Causes Control.

[29] Benamouzig R, Uzzan B, Martin A, Deyra J, Little J, Girard B, Chaussade S (2010). Cyclooxygenase-2 expression and recurrence of colorectal adenomas: effect of aspirin chemoprevention. Gut.

[30] Sadeghi S, Bain CJ, Pandeya N, Webb PM, Green AC, Whiteman DC (2008). Aspirin, nonsteroidal anti-inflammatory drugs, and the risks of cancers of the esophagus. Cancer Epidemiol Biomarkers Prev.

[31] Viswanathan AN, Feskanich D, Schernhammer ES, Hankinson SE (2008). Aspirin NSAID and acetaminophen use and the risk of endometrial cancer. Cancer Res.

[32] Xin B, Yokoyama Y, Shigeto T, Mizunuma H (2007). Anti-tumor effect of non-steroidal anti-inflammatory drugs on human ovarian cancers. Pathol Oncol Res.

[33] Fowke JH, Motley SS, Smith JA, Cookson MS, Concepcion R, Chang SS, Byerly S (2009). Association of nonsteroidal anti-inflammatory drugs, prostate specific antigen and prostate volume. J Urol.

[34] Joosse A, Koomen ER, Casparie MK, Herings RM, Guchelaar HJ, Nijsten T (2009). Non-steroidal anti-inflammatory drugs and melanoma risk: large Dutch population-based case-control study. J Invest Dermatol.

[35] Gee JR, Jarrard DF, Bruskewitz RC, Moon TD, Hedican SP, Leverson GE, Nakada SY, Messing EM (2009). Reduced bladder cancer recurrence rate with cardioprotective aspirin after intravesical bacille Calmette-Guerin. BJU Int.

[36] Kast RE (2007). Melanoma inhibition by cyclooxygenase inhibitors: role of interleukin-6 suppression, a putative mechanism of action, and clinical implications. Med Oncol.

