# Supplementary material for: Nonsteroidal anti-inflammatory drugs using and risk of head and neck cancer: a dose–response meta analysis of prospective cohort studies
Source: Oncotarget. 2017 Oct 5;8(58):99066–74. doi: 10.18632/oncotarget.21524 (PMC5716792; doi:10.18632/oncotarget.21524)
Supplement: Supplementary file 1 [file oncotarget-08-99066-s001.pdf]

Nonsteroidal anti-inflammatory drugs using and risk of head and neck cancer: a dose–response meta analysis of prospective cohort studies

SUPPLEMENTARY MATERIALS

Supplementary Table 1: Sensitivity analysis of the meta-analysis

Deleted one study	Relative risk (95% CI)	*P* for heterogeneity	I^2	*P* for test
Ahmadi et al. (2010)	0.90 (0.83–0.98)	0.182	43.5%	0.013
Becker et al. (2015)	0.85 (0.77–0.94)	0.240	27.5%	0.002
Bosetti et al. (2003)	0.89 (0.82–0.98)	0.034	62.5%	0.012
Di et al. (2015)	0.90 (0.83–0.98)	0.091	60.4%	0.014
Friis et al. (2006)	0.89 (0.83–0.97)	0.177	45.1%	0.002
Friis et al. (2003)	0.86 (0.79–0.95)	0.092	59.5%	0.005
Jayaprakash et al. (2006)	0.82 (0.74–0.90)	0.000	66.3%	< 0.001
Macfarlane et al. (2012)	0.83 (0.76–0.95)	0.296	10.6%	0.002
Macfarlane et al. (2014)	0.90 (0.81–0.99)	0.164	48.5%	0.003
Macfarlane et al. (2015)	0.81 (0.75–0.94)	0.087	53.1%	0.001
Wilson et al. (2013)	0.82 (0.76–0.96)	0.034	62.9%	0.003

Supplementary Table 2: Publication bias analysis of the meta-analysis

	Test	*t*	95% CI	*P*
Total	Begg's test			0.245
	Egger's test	−1.28	−2.12, 0.48	0.211
Aspirin Use	Begg's test			0.215
	Egger's test	−1.01	−2.47, 0.86	0.326
NSAIDs	Begg's test			0.755
	Egger's test	−0.85	−4.03, 1.82	0.415
